# Influence of sports experience on distribution of pro-saccade reaction time under gap condition

**DOI:** 10.1186/s40101-022-00277-1

**Published:** 2022-01-26

**Authors:** Kenji Kunita, Katsuo Fujiwara

**Affiliations:** 1 Department of Sports Instruction, Faculty of Sports and Human, Sapporo International University, 4-1-4-1 Kiyota, Kiyota-ku, Sapporo, 004-8602 Japan; 2 Department of Sports Science, Kanazawa Gakuin University, 10 Sue-machi, Kanazawa, 920-1392 Japan

**Keywords:** Pro-saccade reaction time, Distribution, Gap condition, Overlap condition, Sports experience

## Abstract

**Background:**

Previous studies indicated that substantial individual variation exists in the distribution of pro-saccade reaction times under gap condition. To investigate the influence of sports experience on the distribution, we examined distribution of the pro-saccade reaction time under overlap and gap conditions, for the basketball club, table tennis club, and non-sporting control groups.

**Methods:**

Subjects performed pro-saccade tasks under the overlap and gap conditions, in which the intentional and reflexive disengagement of fixation are important, respectively. Under the overlap condition, the central fixation point was illuminated for a random duration of 1–3 s, then the fixation point was turned off. Just after the switch-off of the fixation point, one of the peripheral targets was illuminated for a duration of 1 s. The visual stimulus under the gap condition was almost the same as that under the overlap condition. However, only the temporal gap between the switch-off of the fixation point and the onset of the target differed between those conditions. The gap duration in the gap condition was set at 200 ms. The mean of median value of the bandwidth showing the earliest peak in the histogram was calculated for each group. Thereafter, for each subject, the bandwidth showing the earliest peak under the gap condition was defined as the criterion bandwidth (0 ms bandwidth). Based on this criterion bandwidth, the mean of the relative frequency was calculated for every 10 ms of bandwidth, for the overlap and gap conditions, in each group.

**Results:**

Under the overlap condition, for all subjects, the pro-saccade reaction times showed unimodal distribution. The means of the median value of the bandwidth showing the earliest peak for the basketball and table tennis groups (approximate 170 ms) were significantly earlier than that for the control group (approximate 190 ms). Under the gap condition, the distribution was bimodal for 11 of 15 subjects in the basketball group and for 5 of 15 subjects in the control group. In the table tennis group, the distribution was not bimodal but unimodal for all 15 subjects. For the basketball group, mean of the relative frequency showed bimodal distribution with approximate 120 ms and 170 ms peaks. For the table tennis and control groups, the mean of the relative frequency showed unimodal distribution with approximate 130 ms and 140ms peak, respectively.

**Conclusions:**

The present study indicated that under the gap condition, the sports experience influenced on the distribution of the pro-saccade reaction time. The pro-saccade reaction time under the condition would show a distinct bimodal distribution for the basketball group and show a distinct and early unimodal distribution for the table tennis group. It was suggested that the physiological factor leading the group difference in the distribution was the effect of sports experience on the disengagement function of fixation.

## Background

The human head weighs approximately 8% of the total body weight. This imposes limits on the gaze accompanying larger and more rapid shift, by using head movements alone [1, 2]. The visual resolution of the retina is highest at the central fovea and decreases exponentially with increasing distance from the fovea [3, 4]. Saccadic eye movement (saccade) that quickly and precisely matches the fovea to the visual object accompanying an abrupt shift is important for perception of the object in various sports [5–9]. Saccades to the appearing visual object are called visually guided saccades or pro-saccades.

The pro-saccade is controlled via supraspinal pathways, including the lateral geniculate nucleus, superior colliculus, reticular formation, occipital cortex, posterior parietal cortex, parietal eye field, and frontal eye field [10, 11]. The functions associated with pro-saccade are detection of the visual target, identification of the target position, saccade preparation, disengagement of fixation, and saccade generation [10, 12]. Disengagement of fixation has been investigated using overlap and gap conditions, in which the timing between switch-off of the central fixation point and lighting of the visual target are operated. The overlap condition is one in which the peripheral visual target emerges before or just after switch-off of the fixation point [13–16]. Under the overlap condition, intentional disengagement of fixation is important for saccade generation, which is particularly related to the frontal eye fields in the higher saccade system [13]. In relation to the involvement of the higher saccade system associated with intentional disengagement, the pro-saccade reaction times under the overlap condition show a unimodal distribution with the peak ranging from 180 to 200 ms [13–15]. On the other hand, the gap condition is one in which the central fixation point is turned off some time (mainly 200 ms) before emergence of the peripheral visual target [13, 15, 16]. Under the condition, the higher saccade system is less involved, and instead, the subcortical pathways and primary visual cortex associated with the reflexive disengagement of fixation are more prominent compared with the overlap condition [13, 17, 18]. Under the gap condition, reaction time shows a bimodal distribution, with the peak at 100–120 ms associated with the reflexive disengagement of fixation (first peak), in addition to the abovementioned peak of 180–200 ms associated with the intensive disengagement of fixation, similar to the situation in the overlap condition (second peak) [13, 17, 18].

However, the previous studies suggested that substantial individual variation exists in the distribution of saccadic reaction times under the gap condition, which shows abovementioned bimodal distribution and unimodal distribution with the peak at 100–120 ms [13, 14, 18]. The individual variation has been investigated from the perspective of learning and training related to the disengagement function of fixation [13, 15, 16]. Sports experience is regarded as one form of learning and training. However, whether the distribution of pro-saccade reaction times under the gap condition is influenced by sports experience has yet to be investigated. To investigate the influence of sports experience on the distributions, we noted that the functions of disengagement of fixation differ markedly between basketball and table tennis. For basketball, attention to the whole visual field and/or parallel attention to various objects including the ball and players in the peripheral visual field are important [19–22]. In particular, intended attention to the visual object with inhibition of reflexive disengagement of fixation to fakes and feints is important. When performing saccade to the appearing visual object in basketball, disengagement of fixation would be intensive. We thus predict that intensive disengagement would be enhanced with increasing experience in playing basketball. We presumed that for the basketball group, the pro-saccade reaction times under the gap condition show a bimodal distribution, consisting of a peak at 100–120 ms associated with the reflexive disengagement of fixation, in addition to the peak at 180–200 ms, associated with the intensive disengagement of fixation. In table tennis, the distance between players is only about 3 m and the playing time in which to return the fast-moving ball is markedly short compared to other ball-sports [6, 23, 24]. Two points in the flight of the ball are crucial for the gaze in table tennis: the beginning of the trajectory and just before the execution of the strike [25]. Because the interval between these two points is approximate 200 ms and markedly short, more intensive disengagement of fixation may result in gaze to the latter point being too late [6, 25]. Therefore, in table tennis, reflexive disengagement of fixation is important. We predict that the reflexive disengagement of fixation would be enhanced with increasing experience in table tennis. Considering these, we presume that for the table tennis group, pro-saccade reaction time under the gap condition shows a unimodal distribution with the peak ranging from 100 to 120 ms, associated with the reflexive disengagement of fixation. Furthermore, we presume that for non-sporting control group, the pro-saccade reaction time under the gap condition shows mixed-type of bimodal and unimodal distribution, because the substantial individual variation exists in the distribution.

In the present study, we examined the distribution of the pro-saccade reaction time under the overlap and gap conditions, for the basketball club, table tennis club, and control groups. By comparing those distributions, we investigated the influence of sports experience on the distribution of the pro-saccade reaction time under the gap condition. The working hypotheses were as follows.

1. The pro-saccade reaction time under the overlap condition would show a unimodal distribution for three groups.
2. The pro-saccade reaction time under the gap condition would show a distinct bimodal distribution for the basketball group and show a distinct and early unimodal distribution for the table tennis group.

## Methods

### Subjects

Subjects were students of Sapporo International University and were divided into three groups. Subjects in the TT group had belonged to the table tennis club for ≥4 years (15 subjects; mean age, 21.2 years). Subjects in the BB group had belonged to the basketball club for ≥4 years (15 subjects; mean age, 20.8 years). Subjects in the C group had never belonged to any sports clubs between elementary school and university (15 subjects; mean age, 20.4 years). No subjects had a history of neurological impairment. In accordance with the Declaration of Helsinki, all subjects provided informed consent after receiving an explanation of the experimental protocol, which was approved by the ethics committee at Sapporo International University.

### Apparatus and data recording

**Fig. 1:**
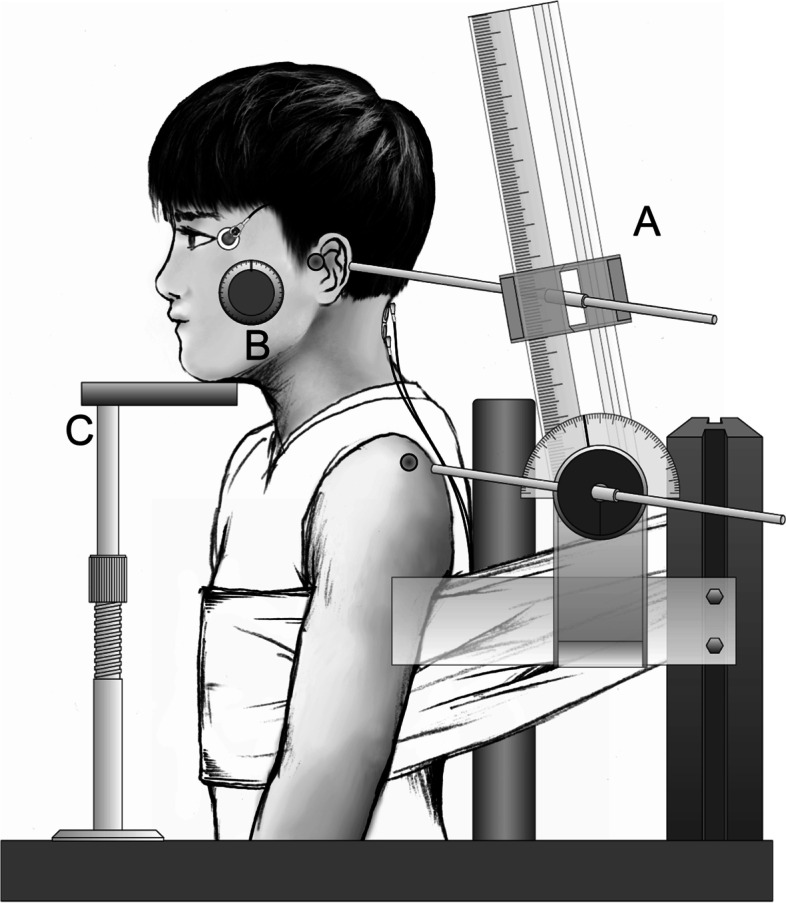
Experimental setup. **A** Neck angle detector, **B** angle detector using the pendulum principle, and **C** chin stand

The experimental setup is shown in Fig. 1. Subjects sat on a steel-frame chair with the back resting against a vertical wall, and the trunk was secured by a cotton band to prevent antero-posterior movement. The knees were kept flexed at approximately 90°, and the feet were rested on a low table. The neck angle was defined as the rotational angle of the tragus around the acromion in the sagittal plane. The angle was at the same angle as the quiet sitting posture, using an angle detector in which the center point was set at the acromion and the distance between the acromion and tragus was regulated. Head inclination angle was determined as the angle between the auriculo-infraorbital line and the gravitational line, and this was maintained at the same angle as the sitting posture to maintain constant sensory inputs from the vestibular organs. An angle detector using the pendulum principle (Level+angle detector; Mitsutomo, Tokyo, Japan) was attached to the temple to confirm this angle. A chin stand was used to support the head and allow maximal relaxation of the neck extensor muscles.

A visual stimulator (LPK-200; Electro Design, Tokyo, Japan) was used to induce saccadic eye movements. Light-emitting diodes (LEDs) located at the central fixation point and at the targets were illuminated for time periods set by the stimulator and a functional generator (WF1966; NF, Kanagawa, Japan). The size of the LED was 0.23° in height and 0.80° wide, with illuminance of 0.02 lumens. LEDs were placed at the height of the nose root, and the distance between the LED at the central fixation point and the nose root was set at 50 cm. Based on Fisher [17] and Braun and Breitmeyer [26], the two lateral targets were located at 4° to the left and right of the central fixation point and were presented with equal probability at random.

The eye movement was measured using electrooculography (EOG). Horizontal EOG was recorded from surface electrodes (P-00-S; Ambu, Ballerup, Denmark) at the outer canthus of each eye, and vertical EOG was recorded from electrodes above and below the left eye. A ground electrode was placed at the center of the forehead. Electrode-input impedance was reduced to < 10 kΩ. The signal from the electrodes was amplified (×2000) using a DC amplifier (AN-601G; Nihon Kohden, Tokyo, Japan). To obtain stable EOG traces, recording began at least 20 min after the placement of electrodes. Surface electrodes with bipolar derivation were used to monitor and record surface electromyography (EMG) for the bilateral upper trapezius muscles. Inter-electrode distance was at about 3 cm. A ground electrode was placed at the spinous process of the C7 vertebra. Electrode impedances were reduced to < 5 kΩ. Signals from these electrodes for trapezius were amplified (×2000) and band-pass filtered (5–500 Hz), using an EMG amplifier (MA1000; DIGITEX, Tokyo, Japan). EOG and visual stimulus data were sent to a computer (Dimension E521; Dell, Kawasaki, Japan) via an A/D converter (ADA16-32/2(CB) F; Contec, Osaka, Japan) at 1000 Hz with 16-bit resolution. EMG data were sent to an oscilloscope (DS-6612; Iwatsu, Tokyo, Japan) to monitor activation.

### Procedure

**Fig. 2:**
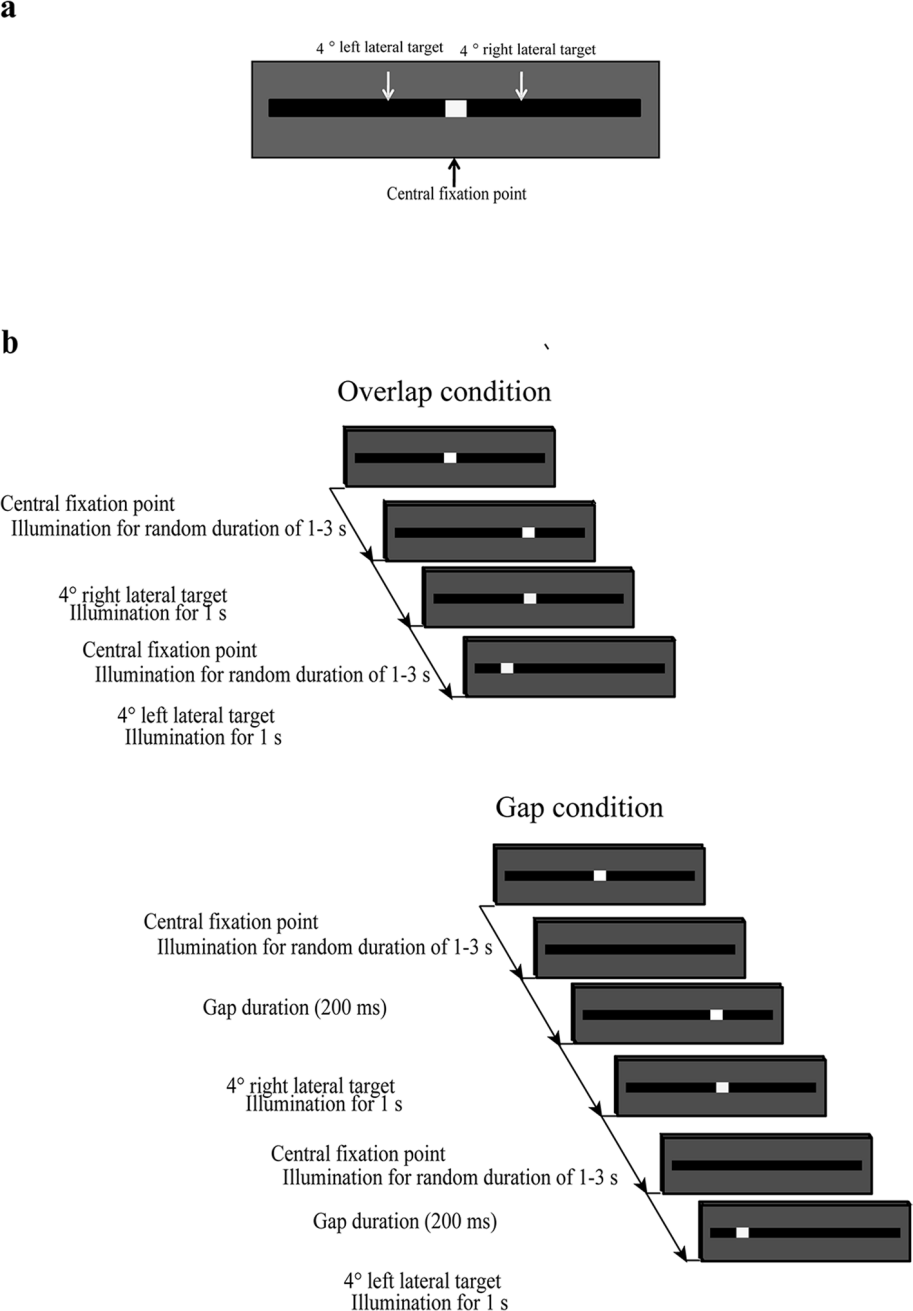
**a** Two lateral targets located at 4° to the left and right of the central fixation point. **b** Presentation protocol in the pro-saccade task

Subjects performed the pro-saccade task under overlap and gap conditions, with instructions to shift the gaze as quickly as possible to the illuminated target (Fig. 2). Initially, to familiarize subjects with the saccade task, 20 practice trials were performed under both conditions. Prior to beginning the task, contraction and relaxation of the trapezius muscles were alternated several times and the subject took deep breaths to relax the muscles. The experimenter verbally instructed the subject to relax the trapezius muscle, which was monitored by EMG. LED illumination was then started. Under the overlap condition, the central fixation point was illuminated for a random duration of 1–3 s, then the fixation point was turned off. Just after the switch-off of the fixation point, one of the peripheral targets was illuminated for a duration of 1 s. The visual stimulus under the gap condition was almost the same as that under the overlap condition, but only the temporal gap between the switch-off of the central fixation point and the onset of the peripheral target differed between those conditions. The gap duration in the gap condition was set at 200 ms. The pro-saccade task was repeated as eight sets of 25 trials; that is, 200 trials were performed in each condition. Rest periods of 3 min and 5 min were provided between sets and between conditions, respectively. The order of those conditions was counterbalanced across subjects to consider potential effects of fatigue and sequential learning.

### Data analysis

**Fig. 3:**
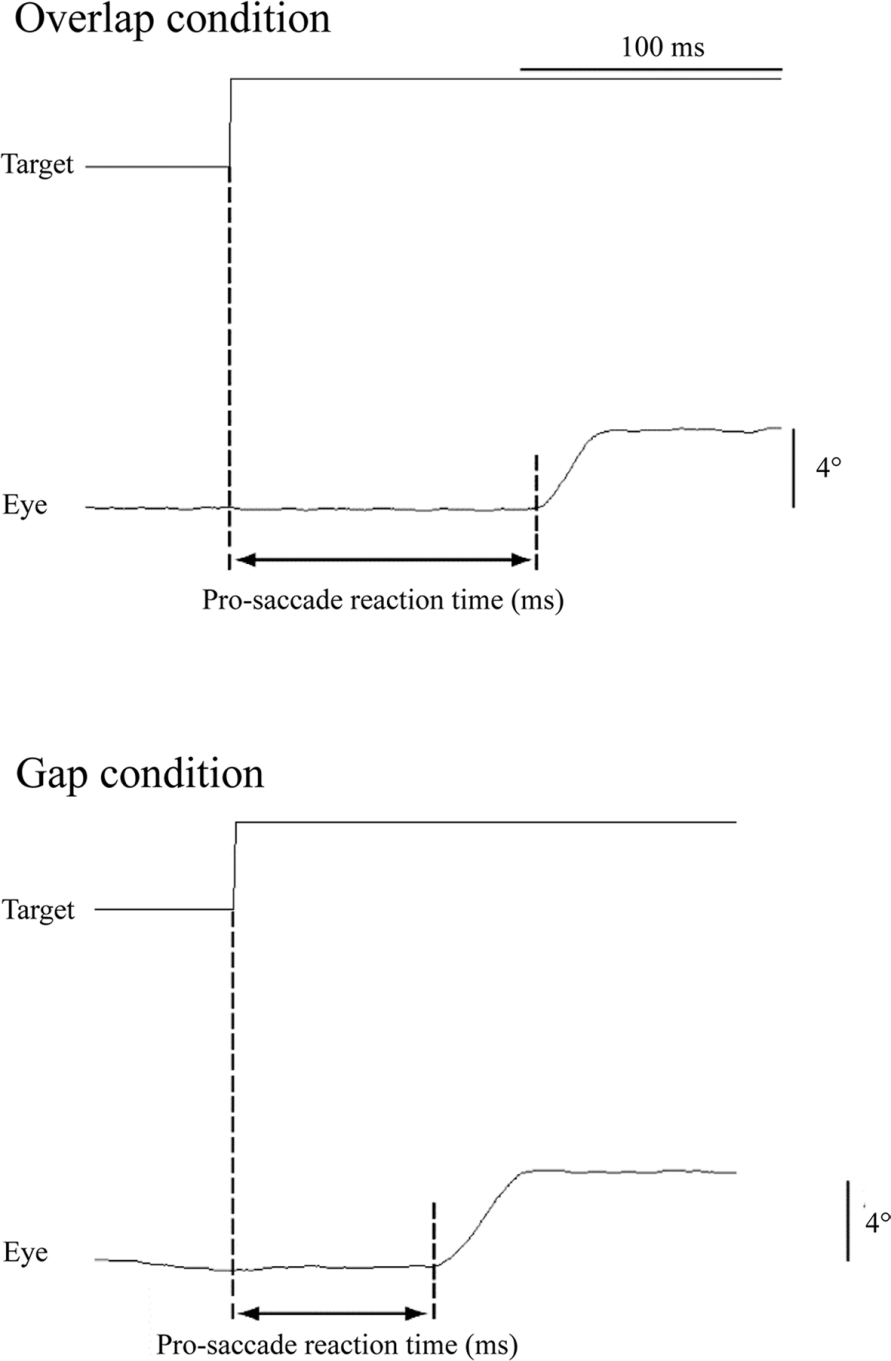
Analysis of pro-saccade reaction time

Analysis of visual stimulus and pro-saccade were performed using BIMUTAS-II software (Kissei Comtec, Matsumoto, Japan). Pro-saccade reaction time was defined as the latency between onset of target movement and beginning of eye movement (Fig. 3). Onset of the eye movement was determined by visual inspection of EOG displacement that was easily discernible from baseline. Referring to Fischer and Weber [13] and Braun and Breitmeyer [26], the histograms of relative frequency for saccadic reaction times were constructed using a bandwidth of 10 ms. The mean and standard deviation (SD) of the median value of the bandwidth showing the earliest peak in the histogram were calculated for each group. Thereafter, for each subject, the bandwidth showing the earliest peak under the gap condition was defined as the criterion bandwidth (0 ms bandwidth). Based on this criterion bandwidth (0 ms bandwidth), the mean and SD of the relative frequency were calculated for every 10 ms of bandwidth, for the overlap and gap conditions, in each group.

### Statistical analysis

The Silverman test using the kernel density method was used for testing the distribution of saccadic reaction times for bi- or unimodality [27–29]. Two-way repeated-measure analysis of variance (ANOVA) was used to assess the significant effects of group and condition on median peak bandwidth in histograms of the relative frequency of saccadic reaction time. When a significant interaction between these effects or a main effect of group was shown, post hoc multiple comparison analysis using Tukey’s honestly significant difference (HDS) was performed to assess differences among groups. Furthermore, one-way repeated-measures ANOVA was used to examine the bandwidths showing no significant difference in comparison to 0 ms bandwidth and the nearest adjacent bandwidths showing a significant reduction from 0 ms bandwidth. To further examine differences suggested by one-way ANOVA, post hoc multiple-comparison analysis using Tukey’s HSD was performed to further examine differences suggested by one-way ANOVA. The alpha level was set at *p* < 0.05. Statistical analyses were performed using IBM SPSS Statistics version 21 (IBM Japan, Tokyo, Japan) and the program of Kusuhashi and Okamoto [30] on the database of the Palaeontological Society of Japan.

## Results

**Fig. 4:**
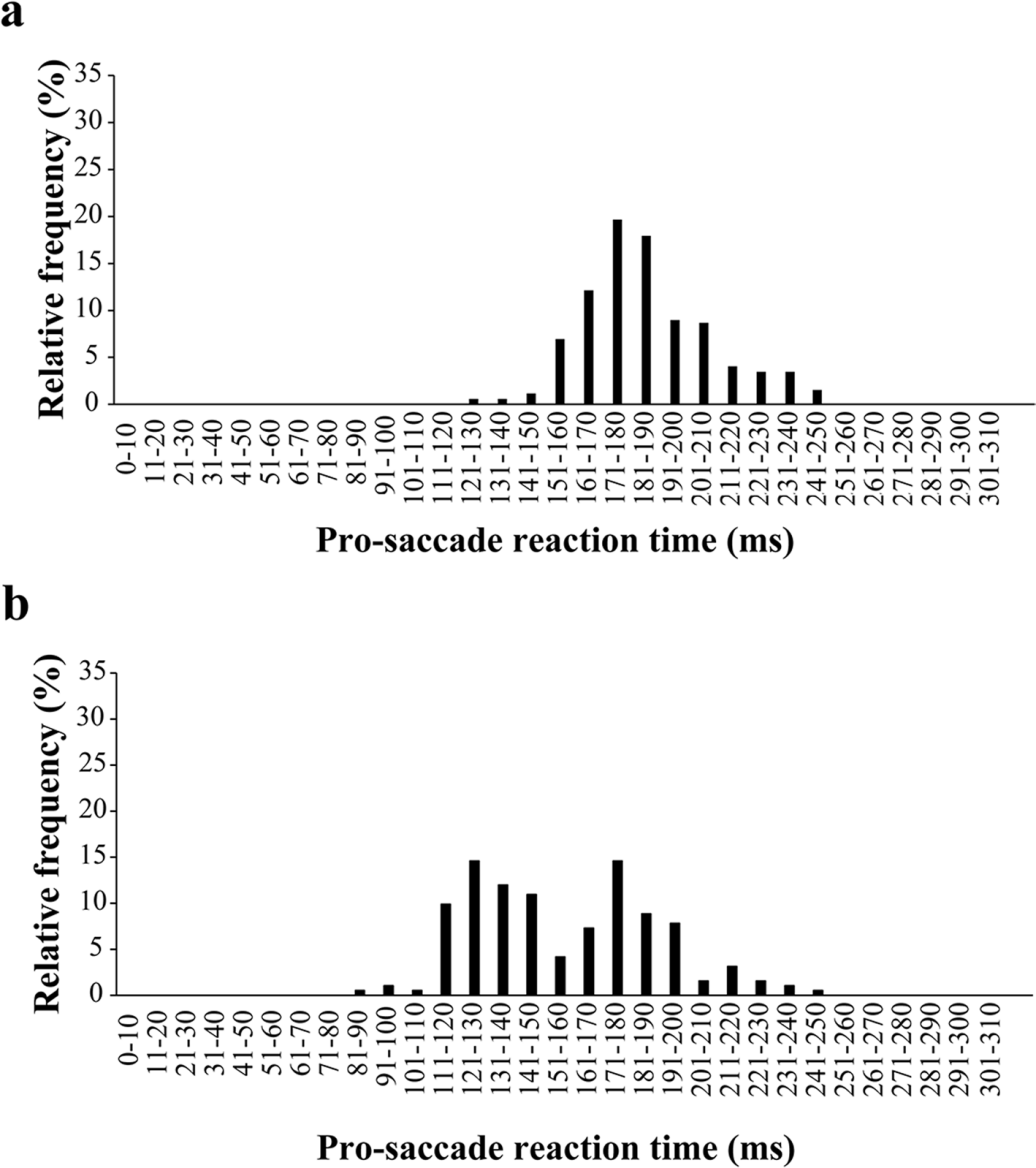
Representative histogram of relative frequency for pro-saccade reaction time. **a** Unimodal distribution under the overlap condition and **b** bimodal distribution under the gap condition

Figure 4 shows representative histogram of relative frequency for the pro-saccade reaction time (**a** unimodal distribution under the overlap condition, **b** bimodal distribution under the gap condition). Table 1 shows the subject numbers which demonstrated the bimodal and unimodal distribution of the pro-saccade reaction time in each group. Under the overlap condition, the distribution of the reaction times was unimodal for all subjects of three groups. Under the gap condition, the distribution was bimodal for 11 of 15 subjects in the BB group and for 5 of 15 subjects in the C group. The distribution in the TT group was not bimodal but unimodal for all 15 subjects.

**Table 1:** Subject numbers which showed the bimodal and unimodal distribution of pro-saccade reaction time in each group

	Overlap		Gap	
	Bimodal	Unimodal	Bimodal	Unimodal
BB group	0/15	15/15	11/15	4/15
TT group	0/15	15/15	0/15	15/15
C group	0/15	15/15	5/15	10/15

**Fig. 5:**
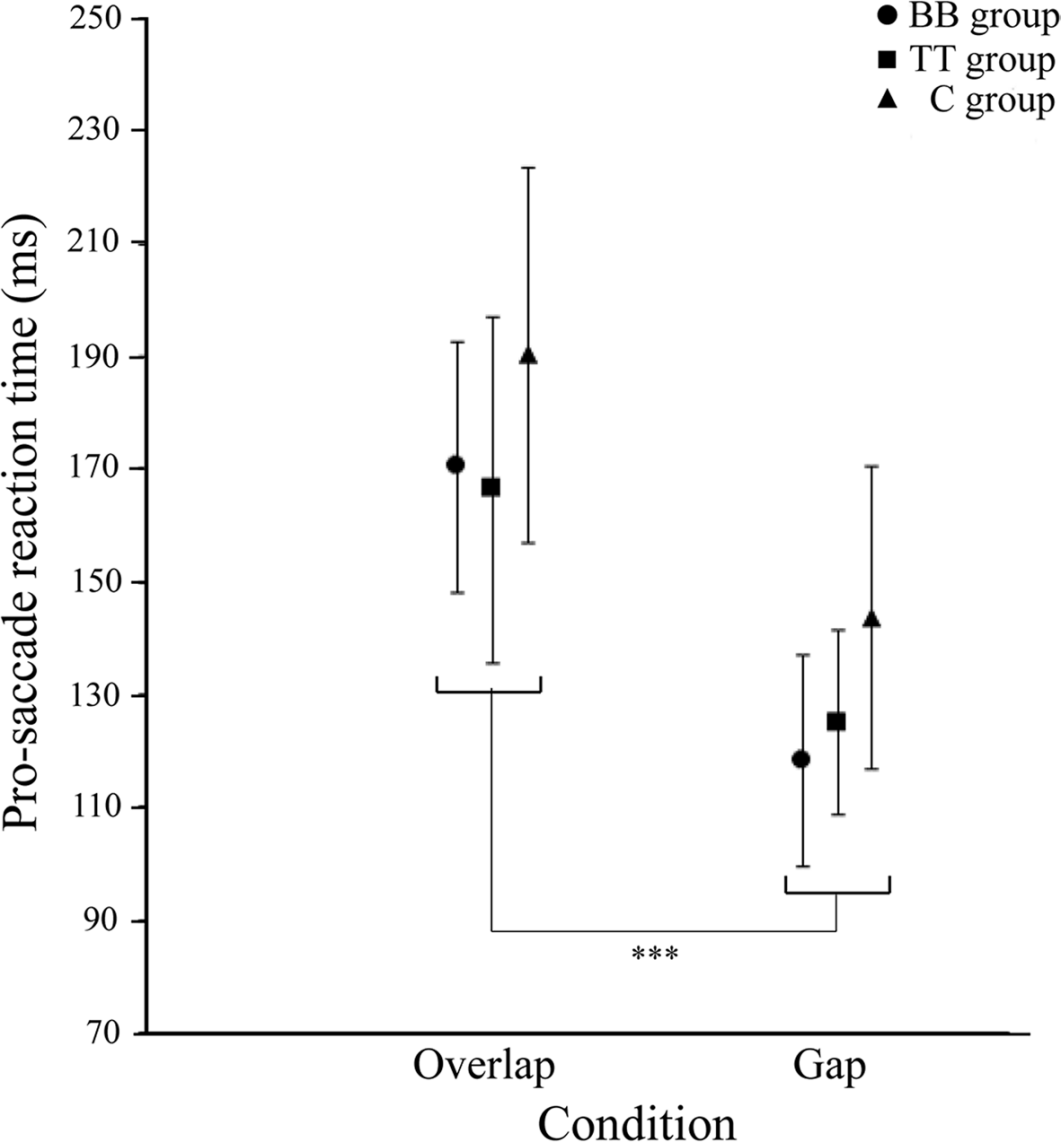
Mean and SD of median value of bandwidth observed for the earliest peak of relative frequency of pro-saccade reaction time. *Significant difference between the overlap and gap conditions

Figure 5 shows mean and SD of median of bandwidth showing the earliest peak in the histogram of the relative frequency for each group. For all groups, the mean value under the gap condition was significantly shorter than that under the overlap condition (overlap condition: BB group 170.7 ± 22.3 ms, TT group 166.3 ± 30.7 ms, C group 190.3 ± 33.4 ms; gap condition: BB group, 118.3 ± 18.8 ms, TT group 125.0 ± 16.5 ms, C group 143.7 ± 27.0 ms; *p* < 0.001). For both conditions, those mean value in the BB and TT groups were significantly shorter than those values in the C group (*ps* < 0.01). Furthermore, no significant group difference in those mean values was found between the BB and TT groups.

**Fig. 6:**
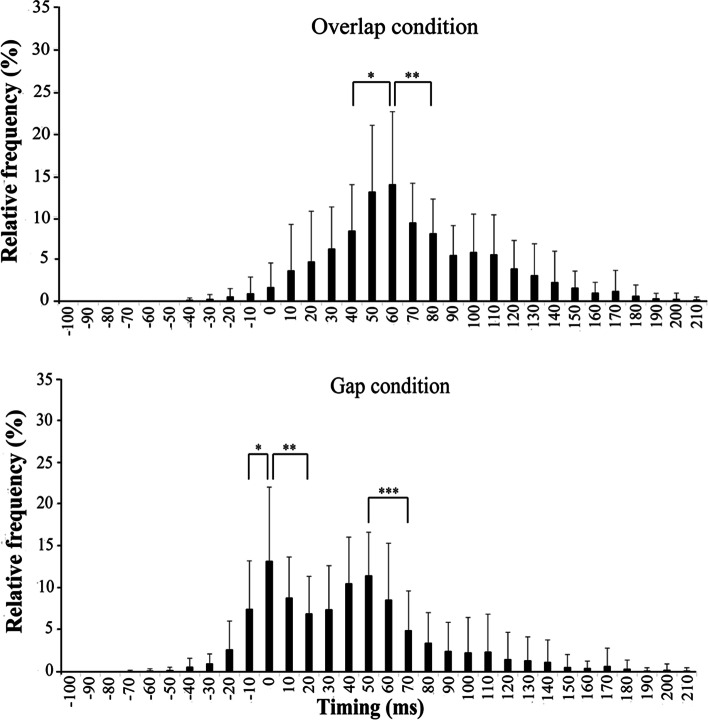
Mean and SD of the relative frequency, based on the criterion bandwidth, for overlap and gap conditions in the BB group. *Nearest adjacent bandwidths showing significant reductions, compared to each peak bandwidth

**Fig. 7:**
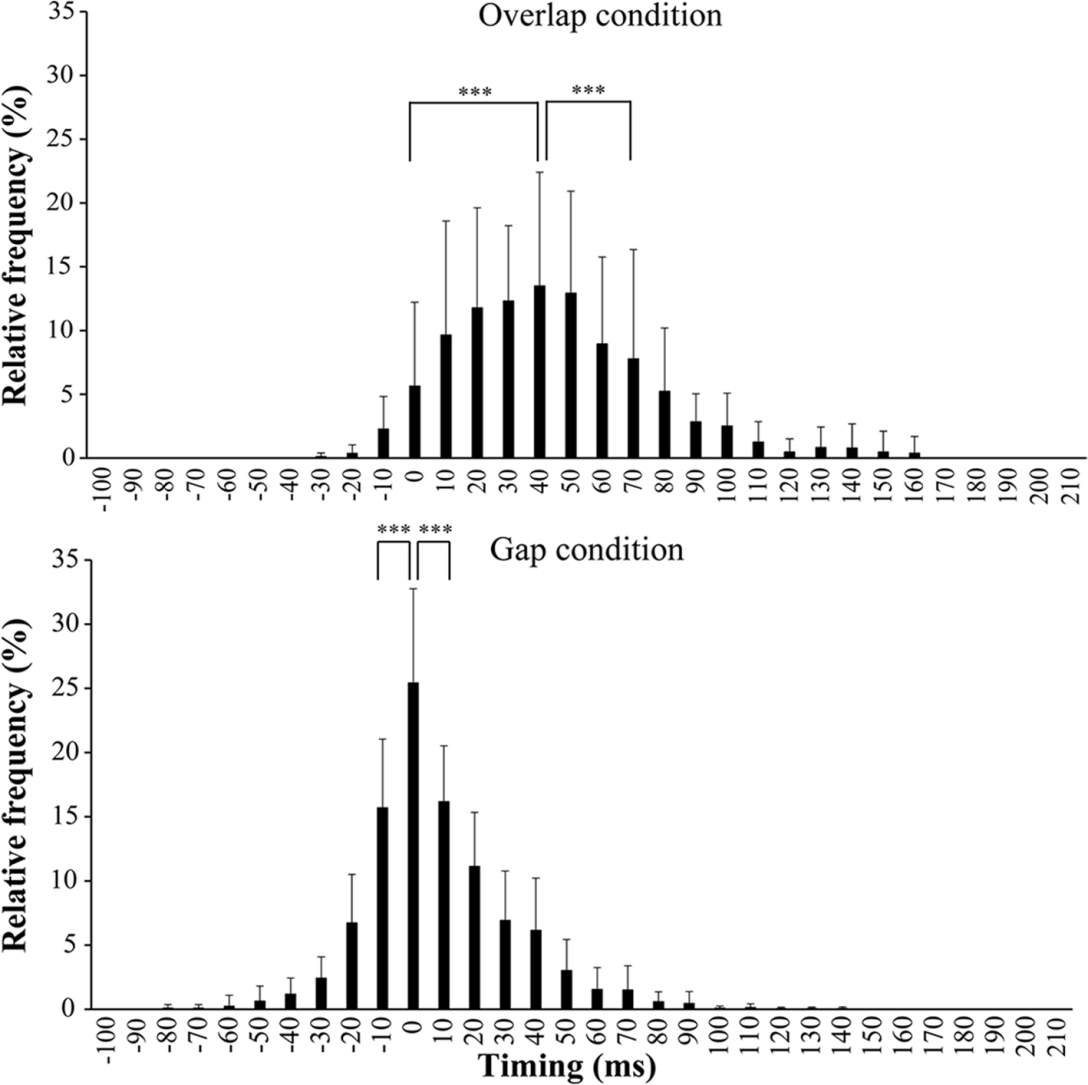
Mean and SD of the relative frequency, based on the criterion bandwidth, for overlap and gap conditions in the TT group. *Nearest adjacent bandwidths showing significant reductions, compared to each peak bandwidth

**Fig. 8:**
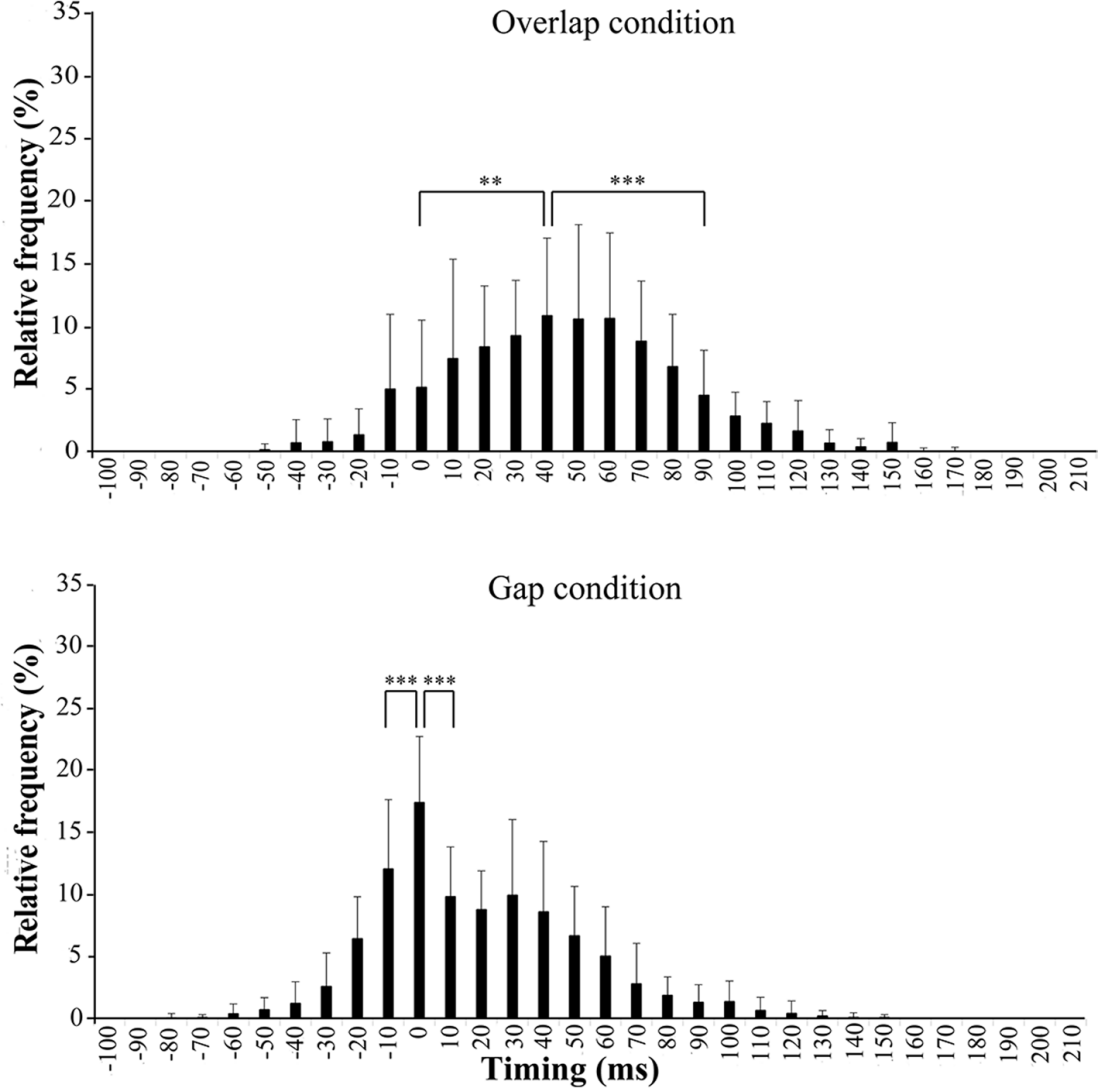
Mean and SD of the relative frequency, based on the criterion bandwidth, for overlap and gap conditions in the C group. *Nearest adjacent bandwidths showing significant reductions, compared to each peak bandwidth

Figures 6, 7, and 8 show mean and SD of the relative frequency, based on the criterion bandwidth (0 ms bandwidth) in each group, for the overlap and gap conditions. For the BB group, under the gap condition, no significant difference was found at +10 ms, +40 ms, and +50 ms bandwidths in comparison to 0 ms bandwidth; namely, bimodal distribution was shown. The nearest adjacent bandwidths showing significant reduction compared to the criterion bandwidth (0 ms; first peak) were −10 ms and +20 ms bandwidths (*ps* < 0.05). Significant reduction was found at +70 ms bandwidth in contrast to +50 ms bandwidth (the second peak; *p* < 0.01). Compared to peak bandwidth under the overlap condition (+60 ms bandwidth), the nearest adjacent bandwidths showing significant reduction were +40 ms and +80 ms bandwidths (*ps* < 0.05). For the TT group under the gap conditions, compared to 0 ms bandwidth, the relative frequency significantly decreased with increasing bandwidth from the criterion bandwidth; namely unimodal distribution was shown. The nearest adjacent bandwidths showing significant reduction were −10 ms and +10 ms bandwidths compared to the criterion bandwidth (0ms bandwidth; *ps* < 0.001). Compared to peak bandwidth under the overlap condition (+40 ms bandwidth), the nearest adjacent bandwidths showing significant reduction were 0 ms and +70 ms bandwidths (*ps* < 0.001). For the C group under the gap condition, compared to 0 ms bandwidth, the relative frequencies in the other bandwidths were significantly smaller (*ps* < 0.001). The relative frequency tended to be decreased with increasing bandwidth from 0 ms bandwidth; however, the frequency at +30 ms bandwidth was slightly larger than that at +20 ms bandwidth. Compared to peak bandwidth under the overlap condition (+40 ms bandwidth), the nearest adjacent bandwidths showing significant reduction were 0 ms and +90 ms bandwidths (*ps* < 0.01).

## Discussion

Pro-saccade under the overlap condition is controlled via the supraspinal pathways, and the frontal eye field is especially important for the intentional disengagement of fixation [10]. Under this condition, pro-saccade reaction time has been reported to show a unimodal distribution with the peak within the range of 180–200 ms [13–15]. In the present study, for three groups, saccadic reaction times under the overlap condition showed a unimodal distribution with the peak within the range of approximate 160–190 ms. Furthermore, median peak bandwidths in the BB and TT groups were significantly shorter than that in the C group. The results in the present study supported the previous studies [8, 31, 32]. Furthermore, no significant group difference in those mean values was found between the BB and TT groups. Considering peak bandwidth, the distribution of reaction time under the overlap condition was likely related to information processing of the higher saccade system associated with the intentional disengagement of fixation.

In previous studies, under the gap condition, it has been suggested that involvement of the higher saccade system is reduced, and that involvements of the subcortical pathways and primary visual cortex associated with reflexive disengagement of fixation are higher, compared with the overlap condition [13, 14, 18]. Therefore, under the gap condition, the distribution of the reaction times shows a bimodal distribution, with the peak in the range of 100–120 ms, associated with reflexive disengagement of fixation (first peak), in addition to the abovementioned peak in the range of 180–200 ms, associated with the intensive disengagement of fixation, which is similar to that for the overlap condition (second peak) [13, 14, 18]. However, in the previous studies, great individual variation was seen in the distribution of the pro-saccade reaction times under the gap condition, which shows abovementioned bimodal distribution and unimodal distribution with the peak at 100-120 ms [13, 14, 18]. The present study found an important finding associated with individual variations under the gap condition. The present study indicated that under the gap condition, sports experience influenced the distribution of saccadic reaction times. A bimodal distribution was seen for 11 of 15 subjects in the BB group and for 5 of 15 subjects in the C group. On the other hand, the distribution in the TT group was not seen bimodal but unimodal for all 15 subjects. For the BB group, the pro-saccade reaction time showed a bimodal distribution with the peaks at approximate 120 ms and 160–170 ms (120 ms + 40–50 ms). For the TT group and C group, the distribution of the reaction times showed a unimodal distribution with the peak at approximate 130 ms and 140 ms, respectively. For the TT group, the relative frequency significantly decreased with increasing bandwidth from the criterion bandwidth. However, for the control group, the relative frequency at +30 ms bandwidth was slightly larger than that at +20 ms bandwidth.; namely the pro-saccade reaction times tended to been shown mixed-type of the bimodal and unimodal distributions. Considering these findings, one of the physiological factors leading the influence of sports experience on the distribution of reaction times under the gap condition may the effect of sports experience on the disengagement function of fixation. As taken into the consideration in the introduction, reflexive disengagement of fixation might be enhanced according to experience with table tennis, and intensive disengagement might be enhanced according to experience with basketball.

For the BB group, under the gap condition, compared to the criterion bandwidth of the first peak, the second peak was found at +40–50 ms bandwidths. Furthermore, from the viewpoint of median peak bandwidth in the histogram, the second peak in the gap condition was similar to the peak in the overlap condition. These results were consistent with previous findings in which the time difference between first and second peaks was approximate 40–50 ms, and no significant time difference was found between the second peak in the gap condition and the peak in the overlap condition [13, 14, 28]. This result indicates that the second peak for the BB group would be related to information processing of the higher saccade system with inclusion of the frontal eye field, associated with intensive disengagement of fixation.

In the summary, the present study indicated that under the gap condition, the sports experience influenced on the distribution of the pro-saccade reaction time. The pro-saccade reaction time under the gap condition would show a distinct bimodal distribution for the basketball group and show a distinct and early unimodal distribution for the table tennis group. It was suggested that the physiological factor leading the group difference in the distribution was the effect of sports experience on the disengagement function of fixation. Furthermore, based on the results in the present study, further investigation was as follows. Under the gap condition, the distribution was not bimodal for 4 of 15 subjects in the BB group and for 10 of 15 subjects in the C group. Further investigation was required on changes in distribution of the pro-saccade reaction time under the gap condition, according to the training with intensive disengagement of fixation.

## Data Availability

The datasets used and/or analyzed during the current study available from the corresponding author on reasonable request.
